# Rhamnolipids Increase the Phytotoxicity of Diesel Oil Towards Four Common Plant Species in a Terrestrial Environment

**DOI:** 10.1007/s11270-012-1190-9

**Published:** 2012-05-17

**Authors:** Roman Marecik, Joanna Wojtera-Kwiczor, Łukasz Ławniczak, Paweł Cyplik, Alicja Szulc, Agnieszka Piotrowska-Cyplik, Łukasz Chrzanowski

**Affiliations:** 1 Department of Biotechnology and Food Microbiology, University of Life Sciences in Poznań, Wojska Polskiego 48, 60-627 Poznań, Poland; 2 Department of Biochemistry, Institute of Molecular Biology and Biotechnology, Adam Mickiewicz University in Poznan, Umultowska 89, 61-614 Poznań, Poland; 3 Institute of Chemical Technology and Engineering, Poznań University of Technology, Pl. M. Skłodowskiej-Curie 2, 60-965 Poznań, Poland; 4 Institute of Food Technology of Plant Origin, University of Life Sciences in Poznań, Wojska Polskiego 31, 60-624 Poznań, Poland

**Keywords:** Bacterial consortium, Diesel oil, Rhamnolipids, Seed germination, Phytotoxicity

## Abstract

The study focused on assessing the influence of rhamnolipids on the phytotoxicity of diesel oil-contaminated soil samples. Tests evaluating the seed germination and growth inhibition of four terrestrial plant species (alfalfa, sorghum, mustard and cuckooflower) were carried out at different rhamnolipid concentrations (ranging from 0 to 1.200 mg/kg of wet soil). The experiments were performed in soil samples with a different diesel oil content (ranging from 0 to 25 ml/kg of wet soil). It was observed that the sole presence of rhamnolipids may be phytotoxic at various levels, which is especially notable for sorghum (the germination index decreased to 41 %). The addition of rhamnolipids to diesel oil-contaminated soil samples contributed to a significant increase of their phytotoxicity. The most toxic effect was observed after a rhamnolipid-supplemented diesel oil biodegradation, carried out with the use of a hydrocarbon-degrading bacteria consortium. The supplemention of rhamnolipids (600 mg/kg of wet soil) resulted in a decrease of seed germination of all studied plant species and an inhibition of microbial activity, which was measured by the 2,3,5-triphenyltetrazolium chloride tests. These findings indicate that the presence of rhamnolipids may considerably increase the phytotoxicity of diesel oil. Therefore, their use at high concentrations, during in situ bioremediation processes, should be avoided in a terrestrial environment.

## Introduction

The use of biosurfactants has been recognised as an environmentally friendly way of enhancing the removal of petroleum products from contaminated soil (Mulligan 2009). However, several studies, concerning the phytoextraction of heavy metals, indicate that rhamnolipids, a well-described group of biosurfactants, may contribute to an inhibition of plant growth (Wen et al. 2010; Gunawardana et al. 2010). A toxic effect of rhamnolipids was observed at the concentration of 2.5 g per 1 kg soil (Wen et al. 2010). Although the application of rhamnolipids contributes to an increased effectiveness of soil flushing processes (at concentrations up to 50 g per 1 kg soil), as well as a higher biodegradation efficiency (at average concentrations below 1 g/l) (Urum et al. 2004; Santa Anna et al. 2007; Makkar and Rockne 2003), the number of studies focused on assessing their effect on phytotoxicity in a petroleum-contaminated terrestrial environment is very limited (Millioli et al. 2009). With this in mind, we investigated the influence of rhamnolipids on the seed germination and growth of four common terrestrial plant species (alfalfa, sorghum, mustard and cuckooflower) in diesel oil-contaminated soil samples. A wide range of concentrations was investigated for both rhamnolipids and diesel oil. Additionally, phytotoxic effects were investigated during a biodegradation of diesel oil, carried out by a bacterial consortium consisting of hydrocarbon degraders, with and without the addition of rhamnolipids. The results of this study may provide an interesting insight into the potential toxicity of rhamnolipids, applied during the bioremediation of petroleum-contaminated soil.

## Materials and Methods

### Chemical Reagents

Petroleum diesel oil (EN 590:2004) was purchased from PKN Orlen, Poland. Rhamnolipids, in a form of a commercially available product JBR-425 (25 % aqueous solution), were obtained from the Jeneil Biosurfactant Company (Saukville, WI, USA). The mixture contains mainly rhamnolipid RL1 (rhamnosyl-β-hydroxydecanoyl-β-hydroxydecanoate) and RL2 (L-rhamnosyl-l-rhamnosyl-β-hydroxydecanoyl-β-hydroxydecanoate). The concentrations of rhamnolipids used during the studies were selected based on their critical micelle concentration (CMC) value (approximately 150 mg/l).

### Microorganisms

The microbial consortium used throughout this study was isolated from a crude oil-contaminated site in Czarna, Poland and exhibited an excellent biodegrading potential towards diesel oil (Owsianiak, Chrzanowski et al. 2009). The diesel-degrading consortium was previously identified and described as belonging to the following bacteria and archaea: *Pseudomonas alcaligenes*, *Ochrobactrum intermedium*, *Sphingobacterium* sp., *Pseudomonas putida*, *Klebsiella oxytoca*, *Chryseobacterium* sp. and *Stenotrophomonas maltophilia* (Owsianiak, Szulc et al. 2009). The consortium did not exhibit the ability to exert any indigenous biosurfactants during this study.

### Plants

The plants used throughout this study belonged to the following species: alfalfa (*Medicago sativa*), sorghum (*Sorghum saccharatum*), mustard (*Sinapis alba*) and cuckooflower (*Cardamine pratensis*). These plant species are commonly used for phytotoxicity tests. The plant seeds were purchased commercially.

### Seed Germination

The phytotoxicity of rhamnolipids, applied in the range of 75–1.200 mg/kg of wet soil, was studied during biodegradation of petroleum hydrocarbons (Makkar and Rockne 2003). Control soil (OECD (Organisation for Economic Cooperation and Development) 1984), used in the germination test (gravimetric water content at 18 %), was acquired commercially (Phytotoxkit tests, Tigret, Warsaw). It was supplemented with diesel oil, at a rising concentration of 0 to 25 ml/kg of wet soil.

The seed germination test was performed in Petri dishes, filled with 50 g of the control soil, with the following sets of experiments: (1) control (seeds in distilled water); (2) control conditions supplemented with rhamnolipids only (at 0, 75, 150, 300, 600 and 1.200 mg/kg of wet soil); (3) seeds germinated in a diesel oil-contaminated soil (at 0, 0.5, 1, 2.5, 5, 10 and 25 ml/kg of wet soil), supplemented with rhamnolipids (at 0, 75, 150, 300, 600 and 1.200 mg/kg of wet soil); and (4) seeds germinated in a diesel oil-contaminated soil (diesel oil at 0, 0.5, 1, 2.5, 5, 10 and 25 ml/kg of wet soil), supplemented with rhamnolipids (at 0, 150 and 600 mg/kg of wet soil) and with the microbial consortium (Owsianiak, Chrzanowski et al. 2009). The control soil was additionally amended with a culture medium, in order to obtain the optimal microbial growth conditions (Owsianiak, Szulc et al. 2009).

After mixing the soil with an appropriate amount of rhamnolipids and diesel oil, a filter paper was transferred onto the soil filled in the Petri dish, and 30 plant seeds were placed on the top of it. Each sample was prepared in three replicates. The dishes were stored in the dark at 25°C. The percentage of germinated seeds (seeds with at least 5-mm long radicles) and radicle length were assessed after 7 days. The germination index was calculated according to these results (Mosse et al. 2010):

$$ {\text{Germination index (GI)}} = \frac{{{G_{\text{x}}}}}{{{G_{\text{c}}}}} \times \frac{{{L_{\text{x}}}}}{{{L_{\text{c}}}}} \times 100\left[ \% \right] $$

where *G*_x_ and *G*_c_ are the number of seeds germinated in the sample and control, respectively, whereas *L*_x_ and *L*_c_ are the length of the radicle in the sample and control, respectively.

### Plant Growth

The plant growth studies were carried out in pots, filled with 500 g of control soil (Tigret, Warsaw), with ten seedlings planted per pot. One uncontaminated soil sample used as a control and other samples were amended with an appropriate amount of rhamnolipids (150 and 600 mg/kg of wet soil) and with diesel oil (0, 0.5, 1, 2.5, 5, 10 and 25 ml/kg of wet soil). Each sample was prepared in three replicates, with a total of 45 pots for each studied plant species. The seedling was grown at an average temperature of 25°C, an average light intensity of 450 μmol s^−1^ m^−2^ and a 16-h photoperiod (Mosse et al. 2010). After 7 days, the plants were destructively harvested and carefully rinsed with redistilled water and the roots and shoots were separated. The tissues were dried for 48 h at 70°C, and the dry matter was assessed.

### Determination of Total Petroleum Hydrocarbon After the Biodegradation

The total petroleum hydrocarbon (TPH) biodegradation was determined according to the procedure described previously by Owsianiak, Chrzanowski et al. (2009).

### Microbial Activity in Soil

The activity of the microbial consortium is reflected by the activity of bacteria dehydrogenases that are able to reduce 2,3,5-triphenyltetrazolium chloride (TTC) to a red product, triphenyl formazan (TPF). The so-called TTC method has been widely used for monitoring the activity of soil microorganisms, and it was recently proposed for screening of the hydrocarbon degraders (Margesin et al. 2000). After the germination test, the soil samples from the Petri dishes (0.5 g) were vortexed with 25 ml of a freshly added mineral medium. A total of 7.5 ml TTC solution (4 mg/ml, in 0.05 M Tris buffer) was added to each sample and then vortexed for 1 min at 2,500 rpm and incubated for 30 min at 30°C. Afterwards, the samples were centrifuged for 15 min at 3,000 × *g*, the supernatant was removed and the soil slurry was vortexed for 1 min with 5 ml of 96 % ethanol and left for 30 min in the dark, for a complete TPF extraction. Since a part of TPF partitioned into residual diesel components, which leads to significant underestimation errors, *n*-hexane was added as a final extraction step (2 ml, 30 min). Absorbance of TPF was measured at 490 nm against *n*-hexane serving as a reference. The activity of dehydrogenases is expressed in micromolar.

## Results and Discussion

### Phytotoxicity

**Fig. 1:**
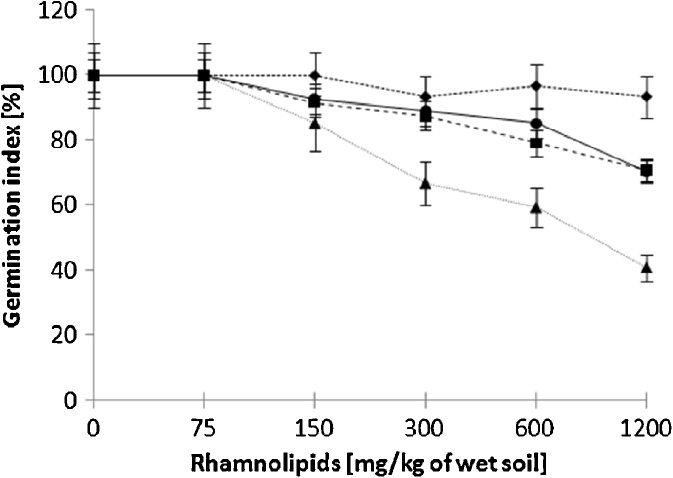
Influence of rhamnolipids on the germination index of four studied plant species, after 7-day germination. Alfalfa is indicated by *circles*, mustard by *squares*, sorghum by *triangles* and cuckooflower by *diamonds*

The initial germination tests were performed without any diesel oil addition, in order to evaluate the toxicity of rhamnolipids towards plants only (Fig. 1). The biosurfactant did not exhibit any phytotoxicity at lower concentrations (75 mg/kg), although an increase in the concentration of rhamnolipids resulted in a decrease of the GI for alfalfa, mustard and sorghum. The decrease of GI was moderate for alfalfa and mustard and most notable for sorghum (GI dropped to 70, 71, and 41 %, respectively). The cuckooflower species remained unaffected.

As demonstrated by Millioli et al. (2009), the sole presence of rhamnolipids may influence the GI of lettuce (*Lactuca sativa*) (Millioli et al. 2009). The authors report that in the case of the latter species, the GI dropped below 50 % at a rhamnolipid concentration of 4 g/kg, decreasing further to 30 % with an increase of the rhamnolipids content (up to 16 g/kg). Interestingly, the presence of rhamnolipids had a much less negative impact on the germination of three out of four plant species tested in our study, at a range of concentrations between 0.75 and 1.2 g/kg. However, the experiments performed by Silva et al. (2010) indicate that cabbage (*Brassica oleracea*) may tolerate the sole presence of rhamnolipids without any significant drop of its GI (Silva et al. 2010). This contributes to a hypothesis that the phytotoxicity of rhamnolipids may be species related.

**Fig. 2:**
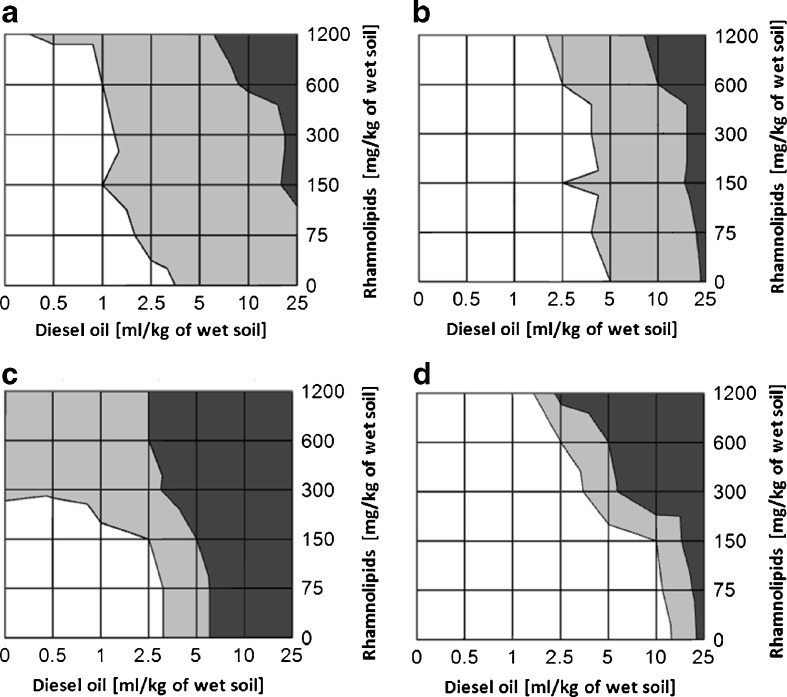
Influence of rhamnolipids and diesel oil on the germination index of four studied plant species (**a** alfalfa, **b** mustard, **c** sorghum, **d** cuckooflower) after 7-day germination. GI = 100–66 % for the *white area*, 66–33 % for the *light grey area* and 33–0 % for the *dark grey area*

Further germination experiments were focused on assessing the phytotoxicity of a diesel oil-contaminated soil, which was supplemented with rhamnolipids (Fig. 2). The combination of the named xenobiotics contributed to a drop of the GI in the case of all studied plant species, which was the most significant at the highest concentration of both compounds. The cuckooflower plants were the most tolerant at low concentrations of rhamnolipids (up to 300 mg/kg) and at higher content of diesel oil (up to 10 ml/kg) (Fig. 2d). In the case of mustard seeds, the GI remained high (66–100 %) at the moderate concentration of diesel oil (up to 2.5 ml/kg) and up to 600 mg/kg of rhamnolipids (Fig. 2b), whereas in this given range of both compounds, the GI of alfalfa is already in the range between 33 and 66 % (Fig. 2a). Interestingly, in the presence of the highest concentration of diesel oil, mustard seeds seem to be 23 less tolerant than alfalfa, even at small amounts of rhamnolipids in the contaminated soil (Fig. 2b), whereas alfalfa’s GI is located in the range below 33 % first at a concentration of approximately 100 mg/kg of rhamnolipids (Fig. 2a). Although the cuckooflower plants display the highest tolerance regarding germination rate towards both diesel oil and rhamnolipids, the decrease of the GI below 33 % is higher when compared with alfalfa and mustard plants.

The decrease of the GI was most notable for sorghum (Fig. 2c), in comparison with the other three species, while the alfalfa and mustard plants exhibited the highest tolerance at the high rhamnolipid and diesel oil concentrations (above 300 mg/kg and 5 ml/kg, respectively). In the samples with a moderate diesel oil contamination (between 1 and 5 ml/kg), notable changes in the GI value occurred at two rhamnolipid concentrations: 150 and 600 mg/kg. Interestingly, the CMC value for a mixture of rhamnolipids is between 50 and 200 mg/l (Chrzanowski et al. 2009; Pornsunthorntawee et al. 2009; Guo et al. 2009). Since the notable changes in the GI value occurred approximately at two rhamnolipid concentrations 150 and 600 mg/kg, where the first concentration belongs to the CMC range and the second is far above it, these two concentrations were chosen for further studies.

**Fig. 3:**
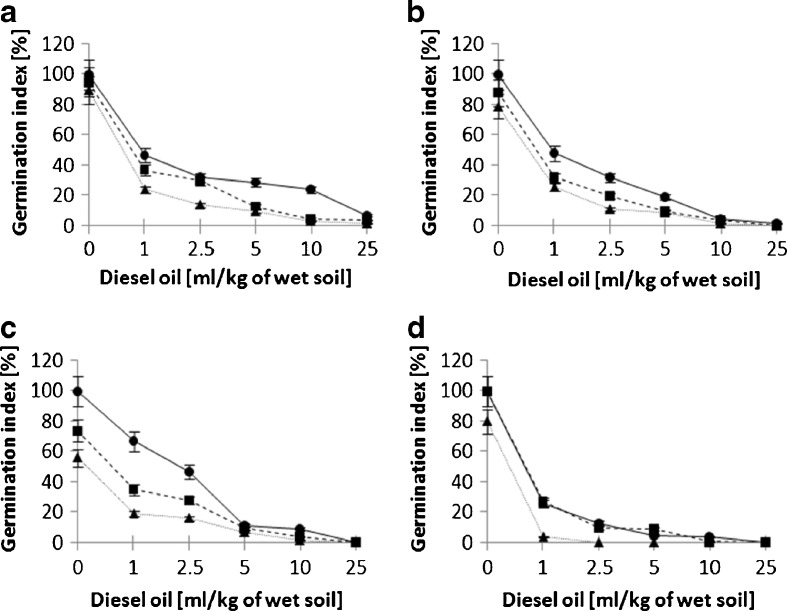
Influence of 7-day long biodegradation of diesel oil and rhamnolipids on the germination index of four studied plant species (**a** alfalfa, **b** mustard, **c** sorghum, **d** cuckooflower); 0 mg/kg of rhamnolipids is indicated by *circles*, 150 mg/kg of rhamnolipids by *squares* and 600 mg/kg of rhamnolipids by *triangles*

The results obtained by Millioli et al. (2009) indicate that the presence of rhamnolipids in a crude oil-contaminated soil (TPH at about 60 ml/kg) results in an increased toxicity towards lettuce, with a complete inhibition of seed germination at 8 g/kg of rhamnolipids (Millioli et al. 2009). As reported by Millioli et al. (2009), it can be concluded that the combination of diesel oil and rhamnolipids may exhibit a notable phytotoxic effect on certain species of plants, even at considerably lower concentrations. This high phytotoxic effect may occur due to the fact that lettuce displays an increased sensitivity towards contaminants, such as petroleum, if compared with millet (*Panicum miliaceum*), radish (*Raphanus* L.), red clover (*Trifolium pratense* L.), and wheat (*Triticum aestivum*) (Banks and Schultz 2005). The aim of the latter experiments was to assess the influence of rhamnolipids on phytotoxicity during a 7-day long diesel oil biodegradation, which was carried out by a bacterial consortium (Fig. 3).

The rhamnolipid-supplemented biodegradation of diesel oil contributed to a further decrease of the GI for all the studied plant species, when compared to the samples with no biodegradation process. The phytotoxicity measured in soil samples after a diesel oil biodegradation process without rhamnolipid supplementation was lower compared to samples containing rhamnolipids at 150 mg/kg for the three studied plant species: alfalfa, mustard and sorghum. The differences were most notable at low concentrations of diesel oil for sorghum and mustard and at higher concentrations for alfalfa. The cuckooflower did not exhibit any differences during the degradation of pure diesel oil and after the addition of the lower concentration of rhamnolipids. However, for each of the studied plant species, their germination index considerably dropped in the samples containing a higher amount of the biosurfactant (600 mg/kg). This effect was most notable for the cuckooflower, and its GI dropped to insignificantly low levels even at the lowest concentration of diesel oil. Based on the obtained results, it can be concluded that the presence of rhamnolipids increases the toxicity of diesel oil in a petroleum-contaminated environment, which results in a significantly increased phytotoxicity.

### Inhibition of Plant Growth

**Fig. 4:**
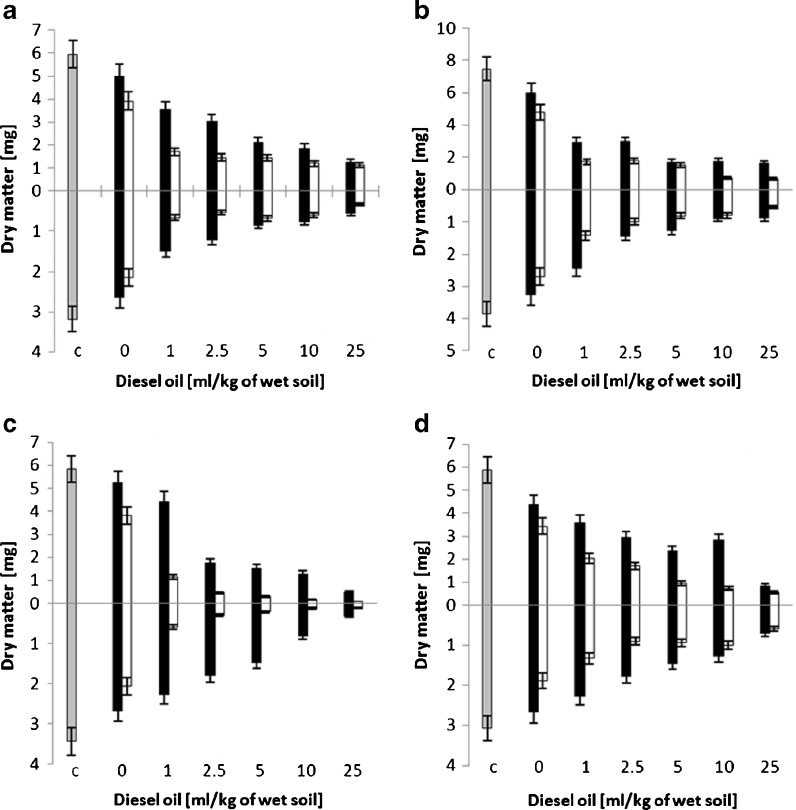
Changes in the shoot (above *x*-axis) and root (below *x*-axis) dry matter of four studied plant species (**a** alfalfa, **b** mustard, **c** sorghum, **d** cuckooflower) after 7 days of growth in soil with diesel oil and rhamnolipids present at different concentrations. Control is indicated by *grey bars*, 150 mg/kg of rhamnolipids by *black bars* and 600 mg/kg of rhamnolipids by *white bars*

Both shoot and root biomass gradually decreased with the increasing concentration of diesel oil for all the studied plant species (Fig. 4). Similar to the results of the initial germination index experiments, the toxic effect was most apparent for sorghum. The use of higher rhamnolipid concentration (600 mg/kg) leads to a considerably higher drop of the dry matter values for both shoot and root compared to the samples containing a smaller concentration of the biosurfactant (150 mg/kg).

### TPH Removal and TTC Activity

**Fig. 5:**
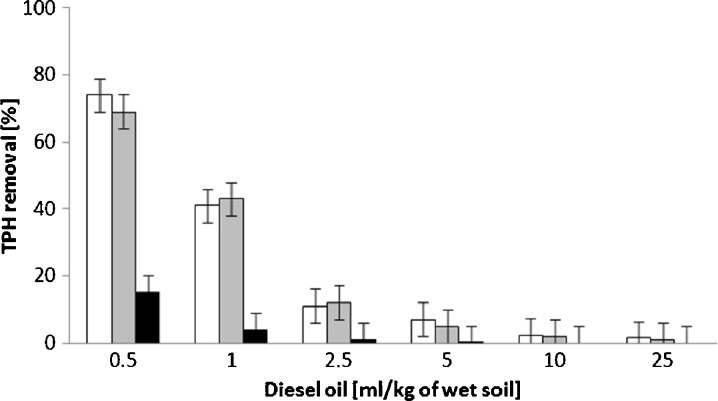
Influence of rhamnolipids on the efficiency of total petroleum hydrocarbon removal after 7 days of biodegradation; 0 mg/kg of rhamnolipids (control) is indicated by *white bars*, 150 mg/kg of rhamnolipids by *grey bars* and 600 mg/kg of rhamnolipids by *black bars*

No significant differences were observed in terms of total petroleum hydrocarbon removal for samples without rhamnolipids and samples with 150 mg/kg of the biosurfactant (Fig. 5). The addition of rhamnolipids at 600 mg/kg resulted in a significant drop of the TPH removal efficiency for samples with a lower diesel oil content. This may have been caused by rhamnolipids being the more preferable carbon source. However, the results obtained during the TTC activity tests seem to contradict with this thesis.

**Fig. 6:**
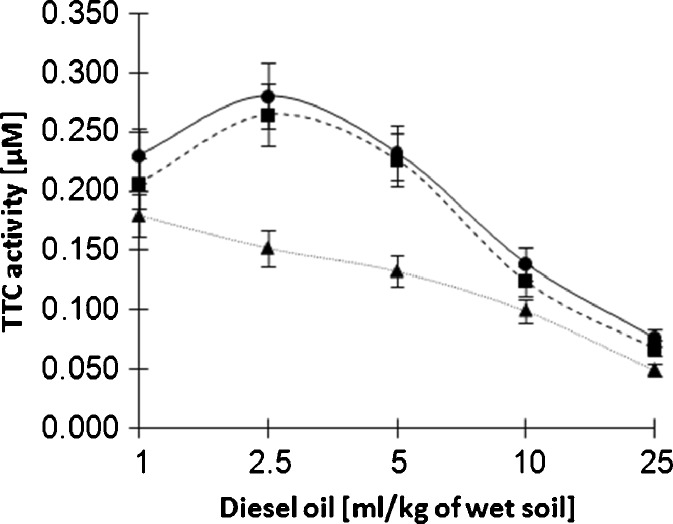
Influence of rhamnolipids and diesel oil present in the soil sample on the measured TTC activity after 7 days of biodegradation; 0 mg/kg of rhamnolipids is indicated by *circles*, 150 mg/kg of rhamnolipids by *squares* and 600 mg/kg of rhamnolipids by *triangles*

The highest microbial activity was observed in the absence of rhamnolipids and at 150 mg/kg of the biosurfactant. As confirmed by our previous studies, the rhamnolipids do not interfere with the microbial growth at a concentration of 150 mg/l (Owsianiak, Szulc et al. 2009); therefore, both curves were very similar (Fig. 6). Again, a notable decrease was observed for samples containing rhamnolipids at 600 mg/kg. At 2.5 and 5 ml/kg of diesel oil, the microbial activity was much higher for samples without rhamnolipids and samples with the lower concentration of the biosurfactant, compared to the samples with a higher rhamnolipid content. The overall activity decreased with the increasing concentration of diesel oil. This may suggest that the rhamnolipids increase the toxicity of diesel oil towards the microbial consortium at a low contamination level. However, Shin et al. reported that even the sole presence of rhamnolipids may be toxic to the microorganisms and result in an inhibited biodegradation of polycyclic aromatic hydrocarbons (Shin et al. 2005).

The progressive decrease of the germination index, plant growth and microbial activity may be caused by changes induced by rhamnolipids in the cell membrane permeability. The fact that the biosurfactant interacts well with diesel oil droplets should also be considered (Chrzanowski et al. 2009), as increasing the water solubility of the otherwise hydrophobic compound may contribute to the increased toxicity as well. It is plausible that both phenomena occur simultaneously, which would result in an overall negative synergy. This effect should be of high priority when considering the use of this biosurfactant for the removal of residual petroleum compounds by soil flushing and biodegradation.

## Conclusions

Our studies confirm that the sole presence of rhamnolipids may potentially be toxic to the natural vegetation. The observed phytotoxicity seems to be species dependent. Furthermore, the addition of rhamnolipids contributes to a significant increase of phytotoxicity in a diesel oil-contaminated soil, and this effect is further enhanced during a rhamnolipid-supplemented diesel oil biodegradation process. Most likely, the biosurfactant interacts with diesel oil particles and enhanced its toxicity towards plants. The obtained results show that the application of rhamnolipids in diesel oil cleanup methods carried out in situ may be limited due to increased phytotoxicity. Future studies will focus on finding an optimal concentration of rhamnolipids, which would be satisfactory in terms of biodegradation effectiveness, removal of residual hydrocarbons and phytotoxicity.
